# President's Address to the Graduates of the Ohio College of Dental Surgery

**Published:** 1867-04

**Authors:** James Taylor

**Affiliations:** President; The Board of Trustees


### PRESIDENT'S ADDRESS TO THE GRADUATES OF THE OHIO COLLEGE OF DENTAL SURGERY, March 6th, 1867.

BY JAMES TAYLOR, M. D., D. D. S., PRESIDENT OF THE BOARD OF TRUSTEES.

*Gentlemen Graduates:*—The Board of Trustees learn with great pleasure that you have pursued your studies with commendable zeal during the session now brought to a close. We are informed that your examinations have been most thorough and highly satisfactory. You are therefore recommended with great unanimity, for the highest honor of this institution.

We know with what commendable pride and often anxiety, this consummation of study is looked forward to; your anxious hours of vigilant toil and study are about to receive their due reward.

You have, gentlemen, now just fairly and honorably mounted the first step on the ladder of professional life. This is *your commencement.* I doubt not, to those of you who may live and practice twenty years, it will appear as the commencement of your studies. You now go forth to test the principles of practice taught in these halls. If you love your profession, and wish to see it make continued progress, put those principles to the most thorough test. If you find error, demonstrate it; your *alma mater* will feel proud of your achievement, and hail you as true sons. Realize, gentlemen, that the foundation of your studies are only laid, and if well laid, a great work has been accomplished. The superstructure is in your own hands. What a world of thought and mind work invites you onward! Think not that Dental science is perfect—that every secret of our complex organization has been revealed—that art has mastered all the perplexities of mechanical Dentistry; that chemistry has fully unlocked all her wonderful store-house of Dental necessities.

There is not, gentlemen, a single department of our science but stands just as inviting for further research as when Hunter commenced his experiments to prove the vitality of the Dental organs. Now, as then, the seeker after truth, sees and must feel that there is a vast amount yet to learn.

The wonders of nature, the harmonies of creative wisdom, the mysteries which envelope the facts of science yet unrevealed; and, we doubt not, full as grand and beautiful as those already revealed. May it not be that we have just cleared away the rubbish which hides the brightest gems of truth, and which shall sparkle in your *crown of rejoicing?*

God has implanted in almost every heart a desire for knowledge—a longing to know more and more of the mysteries of creative power. Every additional gleam of light reveals some new beauty, and thus the lover of science is charmed on and on, ever feeling more compensated for his labor and study by the gratification received. Every department of science is yet rich in hidden treasure. The specialty you have chosen opens a wide field for study. Look at anatomy and physiology—the human system—the most wonderful piece of Mechanism ever devised. Who can understand and explain the human system—the mysterious springs which govern our every action. "Fearfully and wonderfully" are we made!

A life's study would not clear up all the wonderful operations of the varied functions of the body. We take a tooth—what a study it affords! Could we expose a Dental sac at its earliest stage of development, and watch each particle as layer after layer of enamel, cementum, and dentine are so nicely arranged. Could we see that silent, perfect and unerring laboratory of nature at work, as she eliminates and carries to the very spot the lime and other ingredients for this set of organs, we might to some extent, realize the exclamation of the psalmist when he says, "I am fearfully and wonderfully made!"

We might make illustration after illustration, showing in every department future labor. Take one other: Chemists now contend that man is a chemical compound—the teeth decay by chemical action; why then, not replace the carious portion by the aid of chemistry? May we not hope that chemical science will some day enable us to introduce into the cavity new dentine, and cover the same with enamel as perfect in color and durability as the original. From our present stand point we see difficulties in the way. As we ascend higher up the hill of Dental science, these difficulties may vanish and a clear sky open upon us. The road to distinction may already be seen, and we merely wish to incite you up the pathway of knowledge. We fear not the really ambitious. Those *determined to do* will reach a high elevation.

This, gentlemen, is a *diploma* which may truly be considered a certificate of qualification. It, however, confers a right or privilege which is to practice Dental surgery in this

State, and by implication and courtesy, this right is extended all over the United States. I might say all over Europe. Yea, all over the world, for in no quarter can you go but you will find this diploma respected. It dispenses with the examination of a medical board in every country I know of, and generally carries with it an introduction to public confidence. May it ever be thus honored, not only abroad but at home—honored by the recipients, and thus an honor to the Institution by which it is conferred.

Young Gentlemen, the duty now before me is a pleasant one, and it has been rendered more agreeable by the very favorable testimony which the faculty have given of your character and attainments. This is an important and momentous occasion in your lives. Permit me then, to counsel you to be ever diligent in the pursuit of general and professional knowledge.

In accordance now with the authority committed to me as President of the Board of Trustees, I confer on each of you, this Diploma of Doctor of Dental Surgery, and also, with great pleasure, present to you a copy of the Holy Scriptures with the earnest request that you will ever make it "the man of your counsel" to guide you in the path of duty and trial in this life, and that you fail not to attain the blessedness and the glory of the life to come.

Gentlemen graduates, you have now received your sealed credentials. The seal bears this devise: An open book with the motto, "Science combats disease." The book of science is open to you. You are invited to study well its pages. It contains no treasure which the diligent student may not claim.

You now go forth to test your weapons in this great combat; may they ever be directed by those great principles on which our science is based. Thus directed and skillfully used, success will crown your efforts.

There is no department of the healing art requiring more patient perseverance and untiring industry than that you have chosen. There is not in Dental practice a place or corner for the lazy negligent practitioner. Every process of practice, whether in the mouth or laboratory, should be gone through with perfect accuracy. The least omission of duty, the least neglect, may, yes, nearly always *will*, result in failure. The most of our operations may be regarded as the adjustment of art to medical science. You need not only the profound knowledge of the physician, but the artistic taste and intelligent manipulation of the artist.

The honor and dignity of the profession should be sought in all your operations, ever remembering that the profession's character is in your hands, and the more noble and reputable this is made, the more useful you may become.

The terms of success may be sumed up in a very few words: patient perseverance, continued study and a determination to excel. These three characteristics faithfully carried out will overcome every obstacle not insurmountable.

The first difficult operation you have, will likely call into requisition all the patient perseverance you can command, but recollect, that each difficulty mastered is a double success—a success in the operation as well as in the acquisition of knowledge for future use. If this mastery is thorough and perfect, one trouble less lies in your onward progress. Be patient, for your *patient* may need the same virtue. Cross purposes always give trouble in Dental operations. By your perseverance get all the skill you can, and by your kind, courteous, patient treatment of your patients, get all the assistance they can give; this accomplished, and at least half your troubles are ended. The old maxim "that any thing worth doing at all is worth being well done," is especially applicable to Dental practice.

Continued study—the mind cannot stand still, and books are food for the mind; they are to a certain extent, essential to its growth and development. They are made up of the recorded thoughts of the wise, the great, the good, and the learned. It is true we often find the crude speculative theories of the vain and foolish; but even politeness does not require us to consume our time in their perusal. Make books your study; you can then always select for your companions the good and intelligent. It is not necessary, neither is it desirable that you make Dental surgery your only study; yet you will be surprised to find how one department of science is more or less linked with almost every other, so that when you least expect it, you may find some thought, some fact which may be of great importance in your practice. In the broad field of general literature, you will find much to instruct and edify; and here you may take perfect rest and relaxation from your other studies. The wasted hours if gathered up and devoted to good books, would soon give a fund of knowledge of far greater value than the same amount of time spent in the most successful practice. "Idleness is the mother of vice." Use then, all your spare time in the acquisition of knowledge, not forgetting that true knowledge which cometh from the "Father of lights." Let me entreat you, therefore, first of all, study God's word; here is true "knowledge," that which you can rely upon in all the trials and afflictions of life, and which can light up your passage to the grave.

Determination to excel—our success will be somewhat in proportion to our aim in life. He who sits down content with mediocrity, need not expect excellence in his profession; this cannot be attained or *maintained* without persevering labor. The highest seat in *our synagogue* can not long be held by any one who strives not continuously to excel. There is, of late, a great clambering up the ladder of Dental science. You who are just starting, must look up, up all the time, or some more youthful aspirant will climb over your shoulders and gain the ascent far in your advance. Let your aim, therefore, gentlemen, be high. To some extent we hew out our own positions and fortunes. A determined will knows no failure. A strong purpose, with truth and integrity to guide, exerts a wondrous power; the summit of the mount of Dental science has not yet been attained. He who first gains this eminence may well unfurl his banner, and cry "*excelsior, excelsior.*"

Gentlemen graduates, in behalf of the Faculty and Trustees of this Institution, I welcome you as brethren in the cause of Dental Science.

